# Genetic Parameters for Resistance to Non-specific Diseases and Production Traits Measured in Challenging and Selection Environments; Application to a Rabbit Case

**DOI:** 10.3389/fgene.2018.00467

**Published:** 2018-10-16

**Authors:** Mélanie Gunia, Ingrid David, Jacques Hurtaud, Mickaël Maupin, Hélène Gilbert, Hervé Garreau

**Affiliations:** ^1^GenPhySE, INRA, ENVT, Université de Toulouse, Castanet Tolosan, France; ^2^HYPHARM SAS, La Corbière, Roussay, Sèvremoine, France

**Keywords:** heritability, genetics, resistance to disease, farming, rabbit, genetic parameters, genotype-environment interaction

## Abstract

Breeding for disease resistance is a challenging but increasingly necessary objective to overcome the issues with the reduced use of antibiotics and growing concern for animal welfare while limiting economic losses. However, implementing such strategies is a complex process because animals face numerous diseases, and the environments on selection farms differ from those on commercial farms. We evaluated whether selection for resistance to non-specific diseases based on a single visual record in selection (S) and challenging (Ch) environments is possible. Records from 23,773 purebred rabbits born between 2012 and 2016 were used in this study. After weaning (at 32 days of age), 17,712 rabbits were raised in the S environment and 6,061 sibs were raised in the Ch environment. Clinical signs of disease were recorded for all animals at the end of the test, at a single time point, at 70 or 80 days of age. The causes of mortality occurring before the end of the test were also recorded. Three disease traits were analyzed: signs of respiratory disease, signs of digestive disease, and a composite trait (Resist) taking into account signs of digestive, respiratory and various infectious diseases. This latter composite trait is proposed to capture the global resistance to disease. All disease traits were binary, with 0 being the absence of symptoms. Two production traits were also recorded: the number of kits born alive (4,121 litters) and the weaning weight (13,090 rabbits). Disease traits were analyzed with animal threshold models, assuming that traits are different in the two environments. Bivariate analyses were carried out using linear animal models. The heritabilities of the disease traits ranged from 0.04 ± 0.01 to 0.11 ± 0.03. The genetic correlations between disease traits in both environments were below unity (≤ 0.84), indicating genotype by environment interactions. Most of the genetic correlations between disease and production traits were not significantly different from zero, except between the weaning weight and Resist_S, with a favorable correlation of −0.34 ± 0.12. Given these genetic parameters, for the same level of exposure of rabbits to pathogens, the expected response to selection is a reduction of disease incidence of 4–6% per generation.

## Introduction

Breeding for disease resistance is becoming increasingly important to reduce the use of antibiotics and address the growing concern for animal welfare. It also contributes to reducing production costs at both the selection and commercial levels (Phocas et al., 2016). Improving disease resistance by selection is challenging. During their lifetime, animals can face various pathogens, many of which are not always identified. In addition, when the selection environment differs considerably from commercial environments (i.e., the higher biosecurity level of selection environments entails a lower expression of disease) little or no selection pressure is applied on this trait. To implement such selection, there is still a need for phenotypes that can be easily measured, at a reasonable cost (Merks et al., 2012). In rabbits, previous studies have shown that simple health records, measured once on growing animals of the selection nucleus, can be used to improve disease resistance (Eady et al., 2007; Garreau et al., 2012; Gunia et al., 2015). However, it is not known if such selection will be beneficial for maintaining the health and productivity of animals reared in commercial conditions. To address this issue, disease symptoms were recorded in rabbits at Hypharm's facilities in both a selection environment and more challenging environments. The aim of this study was to determine whether selection for resistance to non-specific diseases is possible, and how records from different environments can improve the genetic gains. We first estimated the genetic parameters of disease resistance traits in two contrasting environments and their genetic and phenotypic correlations with the main production traits. Then, we assessed the expected genetic progress for disease resistance for various selection strategies, including records from different environments in the genetic evaluations.

## Materials and methods

This study was carried out in accordance with the national regulations of agriculture in the framework of the selective breeding schemes of the Hypharm breeding company.

### Animals

Data were collected for animals of the AGP77 maternal rabbit line (Hypharm, Roussay, France). Records from 23,773 purebred rabbits born between 2012 and 2016 were analyzed. This line was created from rabbits of the New Zealand breed in 1975. Animals have been selected for litter size and weaning weight (direct and maternal effects) since 2002. In our dataset, does were inseminated every 42 days and kits were weaned at 32 days of age. All rabbits were born and weaned on a nucleus farm, i.e., in a highly bio-secure and controlled environment, where all candidates for selection were further tested. This farm is hereafter referred to as the selection environment (S). After being weighed at weaning, these rabbits were reared in two different environments: (1) the S environment or (2) sib-testing farms, which were 3 farms with less favorable sanitary conditions than the nucleus farm. The sib-testing farms are hereafter referred to as the challenging environment (Ch). Some full sibs and half sibs of the candidates for selection were tested in Ch. The aim of the challenging environment was to mimic the less protected environmental conditions encountered on some commercial rabbit farms. Farms were semi-open rabbit farms with no artificial heating, cooling or ventilation systems. Rabbits of various age classes were reared in the same rooms, and the frequency of veterinary treatments was kept to a minimum. Sick rabbit groups were treated with water medication according to veterinary requirements. Rabbits (mostly males) from every second weaning batch were sent to the Ch environment at weaning. In total, 6,061 rabbits had health records in the Ch environment (5,864 males and 197 females) and 17,712 in the S environment (5,499 males and 12,213 females). The pedigree included 332 sires and 849 dams. A total of 228 sires had kits with health records in both environment, 29 sires in S only and 1 sire in Ch only.

### Traits

Clinical signs of diseases occurring naturally on farms were recorded at a single time point, at the end of the test at 70 or 80 days of age. Very mild clinical signs of diseases were recorded. The most likely cause of death was also recorded after necropsy for rabbits that died between weaning and the end of the test. Disease traits were coded as 0 (absence) or 1 (disorder = morbidity at the end of the test or mortality between weaning and the end of the test). Clinical sign of diseases were not recorded between weaning and the end of the test. Rabbits categorized at healthy (0) at 70 or 80 days of age might have been sick individuals who had recovered. Disorders were further divided into the following categories: (1) digestive disease (Dig), which included diarrhea, bloated abdomen, and any form of digestive symptoms, (2) respiratory disease (Resp) which included nasal discharge, lung lesions, eye infection, wry neck, and (3) non-specific diseases (Resist), which combined Dig, Respi, abnormally low weight, and other clinical signs of infectious origin. The disease traits were treated as separate traits depending on the environment (S or Ch), resulting in a total of 6 disease traits: Dig_S, Resp_S, Resist_S, Dig_Ch, Resp_Ch, Resist_Ch. The production traits were the number of kits born alive per doe (NBA) and the weaning weight (WW), which were exclusively recorded in the S environment. Descriptive statistics of the disease and production traits are listed in Tables 1, 2.

**Table 1:** Total prevalence (in %) of non-specific diseases (Resist), respiratory (Resp) and digestive (Dig) diseases in selection (S) and challenging (Ch) environments from 2012 to 2016^1^.

	**S**	**Ch**
Resist	26	41
Resp	14	24
Dig	11	16

1 *N = 17,712 in S and N = 6,061 in Ch*.

2 *Very mild clinical signs of a disease were taken into account*.

**Table 2:** Number of records (N), mean and standard deviation (Std) for the number of kits born alive (NBA) and weaning weight (WW).

	**N**	**Mean**	**Std**
NBA	4,121^1^	9.92	3.34
WW (g)	13,090^2^	664	102

1 *Number of litters*,

2 *Number of rabbits*.

### Genetic parameters analyses

All traits were analyzed using a restricted maximum likelihood method, with the ASReml 3.0 software (Gilmour et al., 2009). Variance components and heritabilities were estimated using single-trait animal threshold models with a logit link function for the binary disease traits and multiple trait linear animal models for the production traits. Genetic and phenotypic correlations between traits were estimated using multiple trait linear animal models. The models included a random additive polygenic effect for all traits, a random common litter effect for the disease resistance traits and WW, a random maternal environmental effect and a random maternal genetic effect for WW, and a permanent environmental effect to account for the repeated measurement of NBA for does. For the 4% of kits cross-fostered at birth to another doe, the maternal genetic effect, the common litter effect, and the maternal environmental effects were assigned to the adoptive suckling mother. The significance of the fixed effects was determined for each trait using the Wald F statistic, which is similar to an ANOVA (Gilmour et al., 2009). Fixed effects were first tested together, and then a stepwise selection of the significant effects was applied. Significant fixed effects (*P* < 0.05) were maintained in subsequent analyses (Table 3). They were: batch and sex for the disease traits and WW, parity of the dam for WW, and a Ch farms effect for the disease traits measured in Ch. The combined effects of year-season of kitting and parity-physiological status (lactating or not at insemination) were applied for NBA.

**Table 3:** Fixed effects included in the models (x), not significant (NS), or not tested (-) for non-specific diseases (Resist), respiratory (Resp), and digestive (Dig) disease in selection (S) and challenging (Ch) environments, for weaning weight (WW), and number of kits born alive (NBA).

**Effect**	**N of levels**	**Resist_S Resp_S Dig_S**	**Resist_Ch Resp_Ch Dig_Ch**	**WW**	**NBA**
Batch	29	x	x	x	-
Sex	2	x	x	x	-
Ch farms	3	-	x	-	-
Parity of the dam	5	NS	NS	x	-
Weaning age	3	NS	NS	NS	-
Year^*^season of kitting	15	-	-	-	x
Parity^*^physiological status^1^	9	-	-	-	x

1 *Lactating or not at insemination*.

To estimate genetic correlations, we used analysis methods for continuous data, which are not theoretically optimal. The suitable methodology is the threshold model (Gianola, 1982). However, assumption of a continuous distribution for these traits is justified for genetic evaluation and for estimates of genetic correlations with continuous traits (Kadarmideen et al., 2003). Several studies showed that the estimates of heritability or breeding values from linear and threshold models are highly correlated (Matos et al., 1997a,b; Ramirez-Valverde et al., 2001). The difference between these methodologies has been shown to be negligible when the incidence of the binary response was between 25 and 75% (Meijering and Gianola, 1985). Except in the case where fixed effects were added, nested models were compared using the restricted likelihood ratio test. When the model comparison corresponded to a test of parameter on the boundary of parameter space (test of variance different from 0 or test of correlation different from 1), the distribution of this test statistic under the null hypothesis is a 50:50 mixture of $\chi_{q}^{2}$ and $\chi_{q+1}^{2}$distributions (Morrell, 1998), where, q is the number of random effects in the reduced model (residual effect excluded). To obtain the standard error of the heritability, we performed a multivariate sampling approach of the variance components as described by Houle and Meyer (2015) 10,000 times and computed heritability for each sample. Standard deviation of the sampling was then used as an estimation of the standard error of the heritability. In addition, we checked the normality of the distribution obtained. If the hypothesis of normality was not rejected, we then used a Student's *t*-test to compare heritabilities obtained in the S and Ch environments.

### Simulation of breeding schemes

To illustrate the genetic progress that could be obtained for non-specific disease resistance, we tested various breeding schemes including Resist_S or Resist_Ch.

#### General parameters

We used the deterministic simulation program SelAction 2.2 (Rutten et al., 2002) to compare the expected selection responses for breeding schemes including resistance to non-specific disease. SelAction predicts responses to selection on pseudo-BLUP estimated breeding values. In our study, we used the option of discrete generations and 1-stage selection. We simulated a selection nucleus of 140 dams and 35 sires. We assumed 7 progeny per litter with a sex ratio of $\frac{1}{2}$. The selection intensity was 15% for males and 25% for females. Rabbits were selected at 70 days of age. The genetic parameters used for the simulation were those obtained in the first part of the study and the variance components obtained with a linear model for Resist_S and Resist_Ch.

#### Alternative breeding schemes

We compared expected selection responses for fictive breeding objectives including disease resistance in different ways. The four tested breeding objectives were:

HResist_S_Ch = 3 × A_NBA_ + 0.15 × A_WW_direct_ + 0.15 × A_WW_maternal_ - 65 × A_Resist_S_ - 65 × A_Resist_Ch_
  HResist_S = 3 × A_NBA_ + 0.15 × A_WW_direct_ + 0.15 × A_WW_maternal_ - 130 × A_Resist_S_
  HResist_Ch = 3 × A_NBA_ + 0.15 × A_WW_direct_ + 0.15 × A_WW_maternal_ - 130 × A_Resist_Ch_
  HProduction = 3 × A_NBA_ + 0.15 × A_WW_direct_ + 0.15 × A_WW_maternal_

with Ax denoting the true breeding value for trait X. The traits NBA was expressed in number of kits, WW_direct and WW_maternal in g, and Resists_S and Resist_Ch in %. The corresponding weights were given in euros per physical unit of the traits.

The weights did not have any economic meaning, as they were derived by trials and errors based on the desired gain methodology (Brascamp, 1984). Pre-trial simulations with SelAction (Rutten et al., 2002) based on a breeding objective including all traits were run to estimate the genetic gain for various sets of weights. Then, a set of weights was arbitrarily chosen to improve non-specific disease resistance in both S and Ch, while increasing the weaning weight and maintaining a stable NBA, resulting in the HResist_S_Ch breeding objective presented above.

For comparison reason, we also assessed the selection response for a breeding objective including only production traits (HProduction) by using the same weights on the production traits. We studied the indirect expected selection response for each trait, even though it was excluded from the breeding objective, as for instance response in Resist_S and Resist_Ch, when selection occurred for HProduction.

For each breeding objective including Resist_S or Resist_Ch, Resist was recorded with one or the other of the following modalities:

1. With Resist_Ch records: Resist_Ch was recorded on a quarter of the sibs of the selection candidate, while Resist_S was recorded on the selection candidates and on the remaining three-quarters of the sibs.
2. Without Resist_Ch records: all selection candidates and sibs had records for Resist_S only. Resist_Ch was not recorded.

For HProduction, we considered that disease resistance traits were not recorded.

#### Correlations between breeding objectives

The correlations between breeding objectives were calculated as:

$$\begin{array}{l} Covariance\left(H_{i},H_{j}\right)=W_{i}^{T}\times Var\left(A_{i,j}\right)\times W_{j}\\ Correlation\left(H_{i},H_{j}\right)=\frac{Covariance\left(H_{i},H_{j}\right)}{\sqrt{\sigma_{H_{i}}^{2}\times \sigma_{H_{j}}^{2}}} \end{array}$$

with **W**${}_{\text{i}}^{\text{T}}$ being the row vector (1 × n) of the weights of the breeding objective H_i_, **W**_j_ the column vector (m × 1) of the weights of the breeding objective H_j_, Var(**A**_i_,_j_) the genetic variance-covariance matrix (n × m) of the traits in the breeding objective H_i_ and H_j_, and σ2_Hi_ the variance of the breeding objective.

## Results

### Phenotypes

The phenotypes analyzed are shown in Tables 1, 2. The moderately high disease prevalence over the test period (26% for Resist_S and 41% for Resist_Ch) reflects the accurate recording of even the slightest clinical signs of disease. Disease prevalence was higher in Ch than S: 5 percentage points for Dig, 10 percentage points for Resp, and 15 percentage points for Resist. The average NBA was 9.92 ± 3.34 kits, and WW was 664 ± 102 g.

### Genetic parameters of disease resistance traits in selection and challenging environments

The genetic parameters of the disease traits are provided in Table 4. The genetic correlations between environments for each disease trait are given in Table 5. The heritabilities of the disease traits were low, ranging from 0.04 ± 0.01 (Resist_S) to 0.11 ± 0.03 (Dig_Ch). Heritabilities tended to be higher in Ch than in S. However, the difference was not significantly different from zero. The common litter effect also tended to be higher in S (0.05 ± 0.01) than in Ch (0.01 ± 0.01). The genetic and phenotypic correlations between Resist on the one hand, and Dig and Resp on the other hand, were moderate to high, ranging from 0.52 ± 0.13 to 0.74 ± 0.08, and were similar in both environments (Table 5). The phenotypic correlation between Resp and Dig was moderate and negative, while the genetic correlation was negative but not significantly different from zero. The genetic correlations between Ch and S for each disease resistance trait were below unity, demonstrating significant interaction between the genotype and the environment for Resist and Dig, but not for Resp (Table 4). The genetic correlation between S and Ch environments was higher for Resp (0.84 ± 0.12) than for Dig (0.48 ± 0.16), the estimate for the composite trait Resist being intermediate (0.70 ± 0.13).

**Table 4:** Estimates of variance components, heritabilities, common litter effect, and genetic correlations between selection (S) and challenging (Ch) environments for non-specific disease (Resist), respiratory (Resp), and digestive (Dig) disease (± standard errors).

**Environment**	**Resist**		**Resp**		**Dig**	
	**S**	**Ch**	**S**	**Ch**	**S**	**Ch**
σ2_a_	0.15 ± 0.04	0.29 ± 0.08	0.26 ± 0.06	0.31 ± 0.08	0.26 ± 0.07	0.42 ± 0.12
σ2 _com.litter_	0.14 ± 0.03	0.01 ± 0.01	0.19 ± 0.04	0.00 ± 0.00	0.20 ± 0.05	0.04 ± 0.07
σ2_e_	1.00 ± 0.00	1.00 ± 0.00	1.00 ± 0.00	1.00 ± 0.00	1.00 ± 0.00	1.00 ± 0.00
σ2_p_	3.59 ± 0.04	3.60 ± 0.07	3.75 ± 0.06	3.60 ± 0.08	3.74 ± 0.07	3.75 ± 0.10
h2	0.04 ± 0.01	0.08 ± 0.02	0.07 ± 0.02	0.09 ± 0.02	0.07 ± 0.02	0.11 ± 0.03
c2 _com.litter_	0.04 ± 0.01	0.01 ± 0.01	0.05 ± 0.01	0.00 ± 0.00	0.05 ± 0.01	0.01 ± 0.02
r${}_{\text{g}}^{1}$	**0.70** ±**0.13**		0.84± 0.12		**0.48** ±**0.16**	

*$\sigma_{\text{a}}^{\text{2}}$, direct genetic variance; $\sigma_{\text{com}.\text{litter}}^{\text{2}}$, common litter effect variance; $\sigma_{\text{e}}^{\text{2}}$, residual variance; $\sigma_{\text{p}}^{\text{2}}$, phenotypic variance; h^2^, direct heritability; c${}_{\text{com}.\text{litter}}^{\text{2}}$, common litter effect; rg, genetic correlation. Variance components; heritability and common litter effect were estimated with a single-trait animal threshold model (logit transformation). Genetic correlations between S and Ch were estimated with a two-trait linear animal model*.

1 *Genetic correlation values in bold type are significant different form one at P < 0.001*.

**Table 5:** Genetic correlations (above diagonal) and phenotypic correlations (below diagonal) for non-specific disease (Resist), respiratory (Resp), and digestive disease (Dig) in selection (S) and challenging (Ch) environments (± standard errors).

	**Resist_S**	**Resp_S**	**Dig_S**		**Resist_Ch**	**Resp_Ch**	**Dig_Ch**
Resist_S		**0.69** ±**0.08**	**0.66** ±**0.09**	Resist_Ch		**0.74** ±**0.08**	**0.52** ±**0.13**
Resp_S	**0.67** ±**0.00**		−0.06 ± 0.16	Resp_Ch	**0.66** ±**0.01**		−0.19 ± 0.17
Dig_S	**0.60** ±**0.01**	−**0.12** ±**0.01**		Dig_Ch	**0.54** ±**0.01**	−**0.25** ±**0.01**	

*Correlations were estimated with two-trait linear animal models*.

*Values in bold type are significantly different form zero at P < 0.01*.

### Genetic parameters of production traits and correlations with disease traits

The variance component estimates of the production traits are shown in Table 6 and the correlations with the disease resistance traits in Table 7. The heritability of NBA was low (0.16 ± 0.03); the direct heritability of WW was moderate (0.29 ± 0.04) and its maternal heritability was very low (0.05 ± 0.02). The phenotypic correlation between NBA and WW was null. The genetic correlation between NBA and direct effects for WW was negative but not significantly different from zero. On the contrary, the genetic correlation between NBA and WW_maternal was moderate and positive (0.51 ± 0.16). The phenotypic correlations between Resist_S or Resist_Ch and the production traits were negative and low to moderate. Most of the genetic correlations between Resist_S or Resist_Ch and the production traits were not significantly different from 0, except for Resist_S and WW_direct which was negative (i.e., favorable).

**Table 6:** Estimates of variance components, heritabilities, common litter effect, permanent environment effect for the number of kits born alive (NBA) and weaning weight (WW) (± standard errors).

	**NBA**	**WW**
σ2_a_	1.71 ± 0.38	2986 ± 420
σ2_com.litter_		1218 ± 104
σ2_mat.env_		730 ± 140
σ2_perm.env_	0.32 ± 0.23	
σ2_m_		474 ± 168
σ2_e_	8.47 ± 0.21	5620 ± 226
σ2_p_	10.49 ± 0.30	10186 ± 202
h2	0.16 ± 0.03	0.29 ± 0.04
c2_com.litter_		0.12 ± 0.01
c2_mat.env_		0.07 ± 0.01
c2_perm.env_	0.03 ± 0.02	
m2		0.05 ± 0.02
Repeatability	0.19 ± 0.02	

*$\sigma_{\text{a}}^{\text{2}}$, direct genetic variance; $\sigma_{\text{com}.\text{litter}}^{\text{2}}$, common litter effect variance; $\sigma_{\text{mat}.\text{env}}^{\text{2}}$, maternal environment variance; $\sigma_{\text{perm}.\text{env}}^{\text{2}}$, permanent environmental variance; $\sigma_{\text{m}}^{\text{2}}$, maternal genetic variance; $\sigma_{\text{e}}^{\text{2}}$, residual variance; $\sigma_{\text{p}}^{\text{2}}$, phenotypic variance; h^2^, direct heritability; c${}_{\text{com}.\text{litter}}^{\text{2}}$, common litter effect; c${}_{\text{mat}.\text{env}}^{\text{2}}$, maternal environment effect; m^2^, maternal heritability; repeatability, ($\sigma_{\text{a}}^{\text{2}}$ + $\sigma_{\text{perm}.\text{env}}^{\text{2}}$)/ $\sigma_{\text{p}}^{\text{2}}$. All variance component were estimated with three-trait linear animal models*.

**Table 7:** Genetic correlations (above diagonal) and phenotypic correlations (below diagonal) for non-specific diseases (Resist) in selection (S) or challenging (Ch) environments, number of kits born alive (NBA), and the direct and maternal effects of weaning weight (WW) (±standard errors).

	**Resist_S**	**Resist_Ch**	**NBA**	**WW_direct**	**WW_maternal**
Resist_S		**0.70** ±**0.13**	−0.08 ± 0.14	−**0.34** ±**0.12**	−0.06 ± 0.20
Resist_Ch	−		−0.06 ± 0.16	−0.05 ± 0.14	−0.04 ± 0.22
NBA	−**0.38** ±**0.03**	−**0.37** ±**0.02**		−0.22 ± 0.11	**0.51** ±**0.16**
WW_direct	−**0.13** ±**0.01**	−**0.11** ±**0.02**	0.03 ± 0.03		**0.71** ±**0.12**

*Correlations were estimated with three-trait linear animal models*.

*Values in bold type are significantly different form zero at P < 0.05*.

### Expected genetic gain

We compared the selection response for four breeding objectives including Resist_S, Resist_Ch, or both, or only production traits. The breeding objectives were highly correlated, with correlations greater than or equal to 0.75 (Table 8). The correlation was very high between the breeding objectives with Resist_S or Resist_Ch on the one hand and the breeding objective including both traits on the other hand (0.93).

**Table 8:** Correlations between the breeding objectives^1^.

	**HResist_Ch**	**HResist_S_Ch**	**HProduction**
HResist_S	0.75	0.93	0.76
HResist_Ch		0.93	0.78
HProduction			0.77

1 *HProduction, breeding objective including the direct and maternal component of weaning weight and the number of kits born alive*.

HResist_S, HProduction + Resistance for non-specific diseases in the selection environment

HResist_Ch, HProduction + Resistance for non-specific diseases in the challenging environment

*HResist_S_Ch, HProduction + Resistance for non-specific diseases in the selection and in the challenging environment*.

The expected genetic gain is provided in Table 9 for the four breeding objectives and the two recording modalities for Resist_Ch. On average across scenarios, the expected genetic gain per generation expressed in genetic standard deviation was −0.45 for Resist_S, −0.27 for Resist_Ch, −0.02 for NBA, 0.44 for WW_dir, and −0.19 for WW_mat. The selection response was very stable for Resist_S across the breeding schemes that included Resist_S or Resist_Ch in the breeding objective, with values ranging from −4.5 to −4.8%. The selection response increased for Resist_Ch along with the weight given to this trait in the breeding objective. The highest selection response for both Resist_S and Resist_Ch was obtained for the breeding scheme Production + Resist_Ch with records for Resist_Ch, with −4.8% for Resist_S and −5.1% for Resist_Ch. For each breeding objective including a disease resistance trait, we quantified the expected genetic gain obtained using records from the challenging environment. Recording Resist in the challenging environment (on a quarter of the sibs of the selection candidates) always improved the selection response for Resist_Ch for all the breeding objectives. This additional genetic progress led to a reduction of disease incidence for Resist_Ch of 1 percentage point per generation (on average across scenarios), which represents an additional genetic gain of 25% (compared with the scenarios where Resist_Ch was not recorded).

**Table 9:** Expected direct selection responses or correlated responses to selection per generation (10 months) in trait unit for non-specific disease resistance (Resist) in the selection (S) or challenging (Ch) environment, for number of kits born alive (NBA), and for the direct and maternal components of weaning weight (WW) for four alternative breeding objectives^1^ including or not records for Resist_Ch.

	**Breeding schemes**						
**Breeding objective**	**HResist_S**		**HResist_Ch**		**HResist_S_Ch**		**HProduction**
**Records on Resist_Ch**	**Yes**	**No**	**Yes**	**No**	**Yes**	**No**	**No^2^**
Resist_S (%)	−4.6	−4.5	−4.6	−4.7	−4.8	−4.7	−2.6
Resist_Ch (%)	−4.2	−3.6	−5.7	−4.4	−5.1	−4.0	−0.6
NBA (n of kits)	−0.032	−0.033	0.005	0.001	−0.013	−0.017	−0.118
WW_direct (g)	26.345	26.464	16.471	19.222	21.892	23.233	34.990
WW_maternal (g)	−4.708	−4.626	−2.225	−2.429	−3.535	−3.616	−8.720

1 *HProduction, breeding objective including the direct and maternal component of weaning weight and the number of kits born alive*.

HResist_S, HProduction + Resistance for non-specific diseases in the selection environment.

HResist_Ch, HProduction + Resistance for non-specific diseases in the challenging environment.

HResist_S_Ch, HProduction + Resistance for non-specific diseases in the selection and in the challenging environment.

2 *NBA, WW_direct and WW_maternal are the only traits recorded for this breeding scheme*.

The correlated selection response for disease resistance traits was low but still favorable when these traits where not included in the breeding objective and not recorded (−2.6% for Resist_S and −0.6% for Resist_Ch with HProduction). HProduction had the highest genetic gain for WW_direct (35 g), but the lowest for all the other traits. Unfavorable trends were obtained for WW_mat with all scenarios (−4.3 g on average), but they were lower (−2.2 g) for the scheme HResist_Ch with records for Resist_Ch. However, there was less genetic gain for WW_dir with this scenario (16.5 g). NBA was also more stable for this scenario with a very slight increase of the trait (0.005 kits).

## Discussion

Improving resistance to non-specific diseases seems to be possible in both selection and more challenging environments. Resistance to non-specific diseases is a heritable trait in both environments.

### Breeding for disease resistance or tolerance to non-specific diseases?

In this study, we considered that an animal was affected with a disease if it showed at least one clinical symptom of infection at a single time point. Such observations of animals under normal or more challenging production conditions are a simple and direct approach in order to select for genetic resistance. However, the expression of resistance to disease is questionable (Rothschild, 1998). Sick animals without symptoms may have been categorized as healthy (poor sensitivity) while healthy rabbits or recovering rabbits may have been categorized as sick animals (poor specificity). This could lead to an underestimation of heritability (Bishop and Woolliams, 2010). The ≪ true ≫ heritability of disease resistance is likely to be higher than our estimates. Nevertheless, the measure proposed here for Resist is simple, easy to record, can be routinely collected on farms, and seems to be a good proxy for improving the resistance and tolerance to the most common diseases faced by animals in various production conditions. No experimental challenges are required and no particular disease is given priority over another. As emphasized by various authors (Guy et al., 2012; Merks et al., 2012), the main issue for effective genetic selection for disease resistance is the identification of phenotypes that can be easily measured and routinely collected on farms. In our manuscript, we use the terms “selection for disease resistance” in a very general way to qualify the genetic improvement of rabbit health. However, it may include both resistance and tolerance to disease. Host resistance refers to the ability to reduce pathogen replication within a host, whereas, tolerance refers to the ability to reduce the impact of pathogens on host performance without necessarily affecting the pathogen burden (Doeschl-Wilson and Kyriazakis, 2012). Tolerance can also be more broadly assessed against abiotic factors (temperature) or production diseases (Kause and Ødegård, 2012). Our phenotypes were based on observed clinical signs at a definite time. They included no information about the presence of pathogens and the infection dynamic, if any. Recording the pathogen burden seems unfeasible on farms due to the high number of pathogen types and the cost of such analyses when trying to limit production costs. The healthy phenotype in our study could therefore be an expression of resistance (the animals succeed to reduce pathogen replication), tolerance to pathogens (the animal is a carrier of the pathogen but maintains its level of performance without showing clinical signs), or tolerance to metabolic disorders (e.g., noninfectious digestive disorders), or result from the absence of contact with pathogens. It has been argued that disease resistance mechanisms are often pathogen-specific, while tolerance mechanisms that prevent or repair damage may be more host than pathogen specific, and may thus offer generic protection for a range of pathogens (Doeschl-Wilson and Kyriazakis, 2012). In our case, we may be improving tolerance, and we may also be improving resistance by selecting animals with a more efficient innate immune response. As observed by Glass (2012), “distinct host resistance and tolerance traits may be less common than traits that involve elements of both strategies which are likely to have evolved together to overcome infectious threats.”

### Heritability of resistance to non-specific diseases

The heritability of the disease traits was low. Similar heritabilities were found in French paternal rabbit lines (Gunia et al., 2015) with heritabilities ranging from 0.030 ± 0.003 to 0.041 ± 0.004 for disease traits on the underlying scale. Other studies estimated higher heritability on the observed scale for disease traits: 0.12 ± 0.05 for bacterial infections in Australian rabbits (Eady et al., 2007), 0.17 ± 0.09 to 0.30 ± 0.06 for non-specific mortality, respiratory diseases, and epizootic rabbit enteropathy in Spanish paternal rabbit lines (Ragab et al., 2015). We found no genetic correlations between respiratory and digestive diseases. This result confirms previous results obtained in paternal lines (Gunia et al., 2015). Nonetheless, contrary to the previous study in which only the main disease syndrome was recorded, two disease syndromes could be recorded for each animal in the present dataset. This reduced the bias caused by the limited recording of multiple disease symptoms for the same animal. However, if the two main syndromes were from the same kind of disease (either digestive or respiratory), a third syndrome from another kind of disease would not be registered. The absence of genetic correlation could depend partially from this recording method. The composite trait Resist was genetically correlated with Resp and Dig, and the correlations were similar in the Ch and S environments. Resist could therefore be a good indicator for improving general disease resistance and reducing the sensitivity of rabbits to digestive and respiratory diseases.

### Genotype by environment interactions for disease resistance traits

Genotype by Environment (G × E) interactions were demonstrated for Resist and Dig, with genetic correlations significantly below unity between S and Ch. We also observed a scaling effect, with higher genetic variances in Ch than S. They were compensated by lower variances due to common litter environment, leading to very similar total variance between S and Ch. The lower between-environment genetic correlation observed for Dig (0.48 ± 0.16) compared with Resp (0.84 ± 0.12) may be explained by the higher variability of digestive diseases compared with respiratory diseases. Digestive syndromes can be caused by various pathogens and give rise to various diseases (epizootic rabbit enteropathy, coccidiosis, enterotoxaemia, colibacillosis) that differ among challenging environment farms, whereas respiratory syndromes are mainly caused by *Pasteurella multocida* in rabbits. In pigs, G × E interactions have been described for production traits between nucleus and testing farms (Merks, 1989), and even between farms of good health status (Hermesch et al., 2015). Another study in rabbits reported G × E interactions for nonspecific mortality, respiratory diseases, and epizootic rabbit enteropathy between animals fed *ad libitum* and a restricted diet (Ragab et al., 2015) with genetic correlations for the disease traits between the two feeding systems ranging from 0.26 ± 0.09 to 0.68 ± 0.07. In poultry, genetic correlations ranging from 0.78 to 0.82 were observed for footpad dermatitis of broilers reared in two contrasting environments. G × E interactions for disease resistance have also been reported for different times of infection occurring naturally in farms, for example resistance and resilience from low to high worm challenges in sheep (Riley and Van Wyk, 2009). G × E interactions have been observed for reproduction traits during high and low challenge loads (due to natural disease agent and other stressors) in pig farms (Herrero-Medrano et al., 2015), or before and after an outbreak of Porcine Reproductive and Respiratory Syndrome (Lewis et al., 2008).

Various ways to account for G × E interactions in selection have been proposed depending on the magnitude of the genetic correlation of traits evaluated between environments. Robertson (1959) proposed that genetic correlations below the threshold of 0.80 could be considered as having biological importance, with significant reranking of animals occurring across environments. Alternative breeding strategies can therefore be considered. Simulations in dairy cattle have shown that developing specific breeding programs for each environment is interesting when the genetic correlation falls below 0.61 (Mulder et al., 2006). The genetic correlation for Resist_Ch and Resist_S is 0.70 ± 0.13, which means that the application of a common breeding program in both environments to improve Resist could be an appropriate strategy. However, rabbit breeding schemes differ from those for dairy cattle: they are pyramidal (as in poultry and pigs) without progeny testing. Therefore, further research needs to be undertaken to compare the advantages of running common or separate breeding schemes for pyramidal selection.

### Disease resistance and production traits

Estimates of variance components for production traits were generally consistent with the literature (Garcia and Baselga, 2002; Mocé and Santacreu, 2010; Loussouarn et al., 2012; David et al., 2015). The correlations between Resist and the production traits were mostly not significantly different from zero or favorable. To our knowledge, no genetic correlation between disease traits and traits selected in maternal rabbit lines have been reported previously. A study in a paternal rabbit line reported that genetic correlations between disease resistance traits and body weight at 63 or 70 days and carcass yield were not significantly different from zero or favorable (Gunia et al., 2015). However, in their review, Stear et al. (2001) observed that genetic correlations between production and disease resistance traits in livestock can be either favorable or unfavorable. The genetic correlations between production traits and Resist_S or Resist_Ch were very similar, despite different magnitudes of genetic variance for Resist in S and Ch.

### Selection strategies to improve disease resistance across environments

Our aim is to reduce the prevalence of disease through selection for host resistance. All the breeding schemes tests showed that we could expect a reduction of disease incidence of 4 to 6% per generation (for the same level of exposure of rabbits to pathogens). If we assume linear genetic progress for the disease resistance traits, we could expect a reduction of prevalence of 41–31% for Resist_Ch and 26–21% for Resist_Ch over 5 generations. However, as stated by (Mackenzie and Bishop, 1999), predicting the consequences and benefits of selection is a difficult step, because altering the genetics of individual animals affects the epidemiology of the disease at the population level. Indeed, if we select rabbits for resistance to disease, pathogen exposure is likely to change. As the number of susceptible animals in the population decreases, pathogen transmission among rabbits will also decrease. Bishop and Stear (1997) modeled the response to selection for resistance to parasites in sheep and obtained better responses to selection than predicted by quantitative genetic theory due to changes in the epidemiology of the disease.

The genetic progress on disease resistance traits was always favorable, even when disease resistance traits where not included in the breeding objective. This result is due to favorable correlations with the other traits. However, as some genetic correlation between production traits and Resist_S or Resist_Ch were not significantly different from zero, the “true” correlated response on disease resistance may be null if we select for HProduction. Except for the breeding scheme with HProduction, the genetic progress on Resist_S was very stable, probably because the amount of information available was always high. For this trait, records on the individual performances of the selection candidates as well as for a large number of sibs were always available for all scenarios including a disease resistance trait in the breeding objective. The genetic correlation between Resist_S and Resist_Ch therefore enabled good genetic progress for Resist_S even when this trait was not directly included in the breeding objective. This relatively high correlation between Resist_S and Resist_Ch and the higher heritability of Resist_Ch could explain why the scenario “Resist_Ch” gave the highest genetic gain for both Resist_Ch and Resist_S. Recording Resist_Ch always resulted in higher genetic progress for this trait, as could be expected. This finding is in accordance with previous results reported in the literature. Testing half-sibs under commercial conditions is considered as a good option to maintain genetic gain in the presence of G × E (Mulder and Bijma, 2005). Improving disease resistance in both environments is important, due to the high variability of environments on commercial farms (climate, pathogen loads). Some commercial farms apply high biosecurity measures and their environment is similar to the S environment, while on others the conditions are closer to our Ch environments. The breeding objectives presented here were based on the desired gain methodology, where the weight applied to the trait is based on the target genetic progress for the breeding company. The weight given to Resist in our simulations was high. Other scenarios with more balanced weights among traits may have led to less progress for Resist. Another methodology to define the breeding objective would be to consider economic weights and weighting the genetic gain in each environment by the relative importance of that environment (Mulder et al., 2006). These weights could reflect the size of the doe population in each environment. Economic weights have previously been derived for rabbit meat production (Cartuche et al., 2014). Litter size was the trait with the highest economic value, and was considered 5 times more important (expressed per standard deviation) than fattening survival. The discrepancy between the two methodologies can also reflect the strategic choices of different breeding companies.

## Conclusion

Selection on non-specific disease resistance or tolerance using simple observations seems to be feasible. The trait is heritable, and the genetic correlations with the other traits under selection are not significantly different from zero or favorable. G × E interactions exist for this trait between selection and challenging environments. Therefore, recording this trait in both environments results in higher genetic progress. Long-term prediction of the genetic gain is difficult, due to the probable changes in disease epidemiology caused by selection. Quantitative genetics theory predicts a reduction of disease incidence by 4–6% per generation. The true genetic gain is likely to be greater than that predicted on the present study. Such selection could have a major impact on the reduction of antibiotic use and on the improvement of animal welfare. The biological mechanisms underlying non-specific disease resistance are not fully understood yet. Beside classical immune parameters, the gut microbiota has recently emerged has a key regulator of immunity. Further studies using high throughput and deep phenotyping approaches of extreme animals with the highest estimated breeding values for disease resistance and disease sensitivity are needed to unravel the mechanisms in play.

## Author contributions

MG performed the analyses, critically interpreted the data, and prepared the manuscript. HerG made substantial contributions to the analyses and interpretation of data. ID made substantial contributions to the analysis, critically interpreted the data, and reviewed the manuscript. JH and MM designed the experimental protocol and critically interpreted the data. HélG made substantial contributions to the interpretation of data and edition of the manuscript.

### Conflict of interest statement

This study was conducted under a research contract between INRA and Hypharm supervised by HerG. JH and MM are employed by Hypharm. However, the possible conflict of interest did not interfere with the outcome of this paper. The remaining authors declare that the research was conducted in the absence of any commercial or financial relationships that could be construed as a potential conflict of interest.
